# Proteomics profiling for cardiovascular risk prediction: transforming clinical care

**DOI:** 10.1093/ehjacc/zuag077

**Published:** 2026-05-26

**Authors:** Isabella Kardys, Manuel Mayr, Johannes Mair, Nicholas L Mills, Kurt Huber, Study Group on Biomarkers of the ESC Association for Acute Cardiovascular Care, Bertil Lindahl, Jasper Boeddinghaus, Louise Cullen, Lori B Daniels, Ola Hammarsten, Kurt Huber, Evangelos Giannitsis, Allan S Jaffe, Konstantin A Krychtiuk, Martin Möckel, Christian Mueller, Matthias Thielmann, Kristian Thygesen, Johannes Mair, Nicholas L Mills

**Affiliations:** Department of Cardiology, Thorax Center, Cardiovascular Institute, Erasmus MC University Medical Center, Dr. Molewaterplein 40, 3015GD Rotterdam, The Netherlands; National Heart and Lung Institute, Imperial College London, 86 Wood Lane, London W12 0BZ, UK; Department of Internal Medicine III—Cardiology and Angiology, Medical University of Innsbruck, Anichstrasse 35, 6020 Innsbruck, Austria; British Heart Foundation Centre of Research Excellence, University of Edinburgh, SU.226 Chancellor’s Building, Royal Infirmary of Edinburgh, 49 Little France Crescent, Edinburgh EH16 4SU, UK; Medical Private University Burgenland (GmbH), Steinamangerstraße 21A, 7423 Pinkafeld, Austria; Austrian Heart Foundation, Nordbergstraße 15/4/47, 1090 Vienna, Austria

Plasma proteomic biomarkers measured using modern technologies have the potential to provide new insights into the pathogenesis of cardiovascular disease, to facilitate the discovery of novel biomarkers, and to enhance personalized risk assessment, improve disease monitoring, and optimize treatment.

Affinity-based approaches using aptamers or antibodies enable the measurement of more than 10 000 proteins from a single sample. Multiplexed approaches provide broad coverage of the plasma proteome, including low-abundance proteins, and offer high throughput; concordance across platforms is limited.^1^ In contrast, mass spectrometry does not rely on binding reagents and can therefore help resolve inconsistencies between affinity-based platforms. Both provide measurements in relative units, unlike immunoassay approaches, which yield absolute concentrations. By using reference peptides, however, targeted mass spectrometry enables absolute peptide quantification, and in the future affinity-based panels may also offer absolute quantitation.

## Proteomics profiling in heart failure

In heart failure, natriuretic peptides are well-established biomarkers for both diagnosis and prognosis, and the prognostic value of cardiac troponins, growth differentiation factor-15 (GDF15), soluble ST2, and galectin-3 has been demonstrated.

Recent studies have shown that by evaluating a more comprehensive proteomic profile, we may be able to identify additional biomarkers that improve prognostication. In patients with heart failure and a reduced ejection fraction (HFrEF) measurement of over 4000 proteins, using an affinity-based aptamer platform, combined with machine learning, resulted in a prediction model that outperformed clinical risk scores, natriuretic peptides, and high-sensitivity cardiac troponin.^2^ The proteomic prediction model included both previously identified and novel proteins and demonstrated the added prognostic value of serial measurements, which better capture the dynamic nature of heart failure. In the same study, cluster analyses identified four dynamic proteomics-based phenotypes, each linked to specific clinical characteristics and prognosis.^3^ Moreover, these phenotypes represented distinct biological processes and could thus provide a step towards more individualized treatment decisions.

## Proteomics profiling in atherosclerosis

The use of protein signatures and machine learning from affinity-based approaches can also improve prediction of myocardial infarction and stroke, compared with clinical models alone.^4^ However, some key proteins in the causal pathway for atherosclerosis, such as apolipoproteins, cannot be reliably quantified by affinity-based approaches.^5^ Apolipoproteins are embedded within lipoprotein particles where epitope masking and complex formation can impair binding. In the UK Biobank, ApoB measured by an affinity-based proteomic platform showed very poor correlation with clinically measured ApoB (*r* = 0.07), resulting in no association with cardiovascular events.^6^ This suggests that biologically and clinically relevant targets may be overlooked when relying solely on affinity-based measurements. Furthermore, two-thirds of individuals with high lipoprotein (a) were misclassified by affinity-based proteomics. Complementary mass spectrometry-based techniques or validated clinical assays are needed to accurately measure apolipoproteins. Precision and the measurement of absolute values are required to guide the initiation and monitoring of treatment response to novel apolipoprotein-lowering therapies.

**Figure 1:**
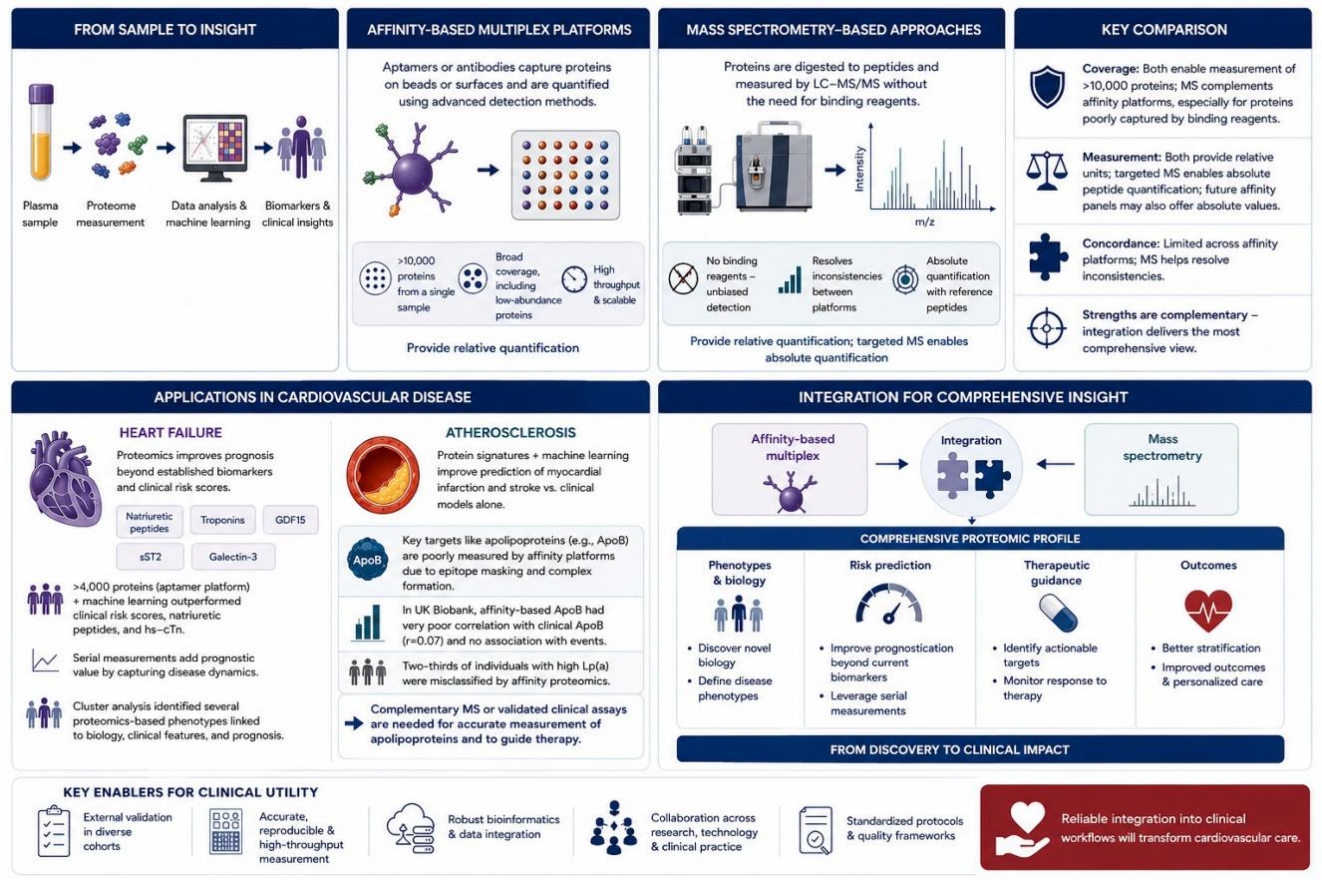
Proteomics profiling in cardiovascular disease.

In summary, recent advances in measuring the plasma proteome together with new bioinformatics approaches hold great promise for scientific discovery and prognostication in cardiovascular disease.^7^ Harnessing these advances to improve care will likely require the integration of different technologies to enable the quantification of both low- and high-abundance proteins (*Figure 1*). Proteomic biomarkers measured using mass spectrometry and multiplex assays can provide valuable new insights into cardiovascular disease phenotypes, progression, and outcomes. However, to fully realize their clinical utility, several key challenges remain. These include the need for further external validation through accurate, reproducible, and high-throughput methods that are both accessible and scalable. Addressing these challenges will require continued collaboration across research, technology, and clinical practice to develop standardized protocols and ensure that proteomic tools can be reliably integrated into clinical workflows, ultimately enabling the transformation of cardiovascular care.

## Data Availability

No new data were generated or analysed in support of this research.

## Appendix

Members of the Study Group on Biomarkers of the ESC Association for Acute Cardiovascular Care:

Bertil Lindahl, MD^1^; Jasper Boeddinghaus, MD^2^; Louise Cullen, MD, PhD^3^; Lori B Daniels, MD^4^; Ola Hammarsten, MD, PhD^5^; Kurt Huber, MD^6,7^; Evangelos Giannitsis, MD^8^; Allan S. Jaffe, MD^9^; Konstantin A. Krychtiuk, MD, PhD^10^; Martin Möckel, MD^11^; Christian Mueller, MD^2^; Matthias Thielmann, MD^12^; Kristian Thygesen, MD^13^; Johannes Mair, MD^14^; Nicholas L Mills, MD, PhD^15^

^1^Department of Medical Sciences, University of Uppsala, Uppsala, Sweden

^2^Cardiovascular Research Institute Basel and Department of Cardiology, University Hospital Basel, University of Basel, Switzerland

^3^Emergency and Trauma Centre, Royal Brisbane and Women’s Hospital, Brisbane, Australia

^4^Department of Medicine, Sulpizio Cardiovascular Center, University of California, San Diego; La Jolla, CA, USA

^5^Department of Clinical Chemistry and Transfusion Medicine, University of Gothenburg, Gothenburg, Sweden

^6^Medical Private University Burgenland (GmbH), Pinkafeld, Austria

^7^Austrian Heart Foundation, Vienna, Austria

^8^Department of Cardiology, University Heidelberg, Germany

^9^Departments of Cardiology and Laboratory Medicine and Pathology, Mayo Clinic, Rochester Minnesota, USA

^10^Department of Internal Medicine II, Division of Cardiology, Medical University of Vienna, Vienna, Austria

^11^Department of Emergency Medicine, Charité-Universitätsmedizin Berlin, Germany

^12^Klinik für Thorax-und Kardiovaskuläre Chirurgie, Westdeutsches Herz- und Gefäßzentrum Essen, University of Duisburg-Essen, Germany

^13^Department of Cardiology, Aarhus University Hospital, Denmark

^14^Department of Internal Medicine III—Cardiology and Angiology, Innsbruck Medical University, Innsbruck, Austria

^15^British Heart Foundation Centre of Research Excellence, University of Edinburgh, Edinburgh, UK
